# Molybdenum Dichalcogenides for Environmental Chemical Sensing

**DOI:** 10.3390/ma10121418

**Published:** 2017-12-12

**Authors:** Dario Zappa

**Affiliations:** Sensor Laboratory, Department of Information Engineering (DII), Università degli Studi di Brescia, Via Valotti 7, 25123 Brescia, Italy; dario.zappa@unibs.it; Tel.: +39-030-371-5767

**Keywords:** transition metal dichalcogenides, chemical sensors, air quality, molybdenum dichalcogenides, molybdenum sulfide

## Abstract

2D transition metal dichalcogenides are attracting a strong interest following the popularity of graphene and other carbon-based materials. In the field of chemical sensors, they offer some interesting features that could potentially overcome the limitation of graphene and metal oxides, such as the possibility of operating at room temperature. Molybdenum-based dichalcogenides in particular are among the most studied materials, thanks to their facile preparation techniques and promising performances. The present review summarizes the advances in the exploitation of these Mo*X*_2_ materials as chemical sensors for the detection of typical environmental pollutants, such as NO_2_, NH_3_, CO and volatile organic compounds.

## 1. Introduction

Transition metal dichalcogenides (TMDs) are a very recent class of materials that are attracting brand new interest in the scientific community. Thanks to the popularity of nanosized carbon-based materials, especially carbon nanotubes (CNTs) and graphene [1,2,3], many efforts have been spent in the past years on exploring materials which can be easily downsized to 1D and 2D configurations. Metal oxide nanowires, nanoflakes and nanotubes, as well as core–shell and other fancy heterostructures [4,5,6], have been fabricated with the intent of enhancing the performance of their respective bulk materials. Among 2D structures, TMDs are becoming very popular due to their abundance and very easy preparation techniques.

**Figure 1:**
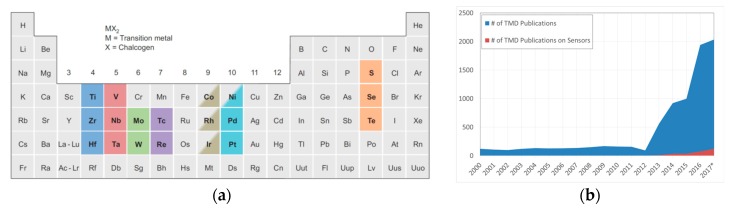
(**a**) Periodic table with highlighted the transition metals and chalcogen elements (S, Se and Te) that form crystalline in 2D layered structures. Co, Rh, Ir and Ni are partially highlighted because only some dichalcogenides form layered structures. Reprinted by permission from Macmillan Publishers Ltd.: Nature Chemistry [7], copyright (2013); (**b**) Number of publications per year on TMDs (in blue) and on TMD-related sensing devices (in red), calculated from fully highlighted materials reported in Figure 1a. * The 2017 data are partial (source SCOPUS, Elsevier B.V.: Amsterdam, The Netherlands).

TMDs can be easily described by the chemical formula *MX*_2_, where *M* is a transition metal from groups 4–10 of periodic table (such as Mo, W and V) and *X* is a chalcogen element (S, Se and Te). In particular, TMDs composed by elements highlighted in Figure 1a have the peculiar property to crystallize in ultrathin layers, leading to the formation of single-layered 2D materials. Therefore, TMDs share some structural similarities with graphene, but they also exhibit some complementary properties and features, making them more appealing from application point-of-view. A typical example is the fabrication of electronic transistors: although graphene has remarkably high carrier mobility at room temperature (more than 15,000 cm^2^/V∙s [3]), it has a poorly-defined bandgap, thus it is difficult to turn the transistor to *off* state. Clearly, it is not well suited to fabricate logic devices in its pristine form. On the contrary, many TMDs are semiconductors, such as MoS_2_, MoTe_2_ and WS_2_; have a wide range of possible bandgaps; and are better suited for their use as an electronic device.

According to SCOPUS data, starting from 2012, there was a huge increase in the total number of TMD-related publications, probably due to the “graphene effect” of 2010 Nobel prize [8] that has shifted the scientific focus towards 2D ultrathin materials (Figure 1b, SCOPUS data, Elsevier B.V.). Nevertheless, the exploitation of TMDs for the manufacturing of sensor devices is still almost unexplored. According to the data, only less than 4% of total TMDs documents indexed by SCOPUS database reports sensor applications based on these materials. However, the trend is positive, so it is reasonable to expect an increase of sensor exploitation as soon as the study of these materials goes further.

**Figure 2:**
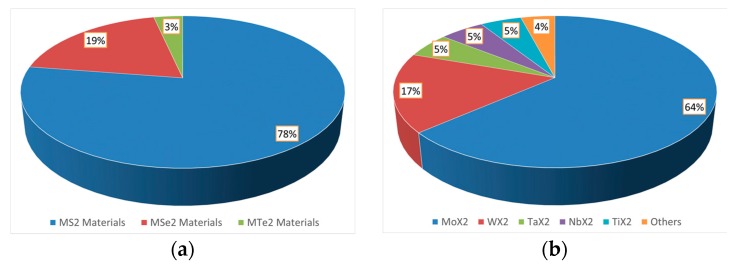
(**a**) Percentage of *M*S_2_ (in blue), *M*Se_2_ (in red) and *M*Te_2_ (in green) manuscripts; and (**b**) chart reporting the percentage of most common transition metals investigated as TMDs. (source SCOPUS, Elsevier B.V.: Amsterdam, The Netherlands).

Looking at the data in detail, it resulted that scientific research is mainly focused on transition metal disulfides (*M*S_2_) (Figure 2a). This is not surprising: MoS_2_ and WS_2_ are by far the most investigated TMDs, with MoS_2_ alone responsible of more than half of total transition metal dichalcogenides publications. Overall, molybdenum dichalcogenides (Mo*X*_2_) are the most studied group, followed by tungsten-based ones, as reported in Figure 2b. 

Among the wide range of existing sensing devices, chemical sensors deserve a special mention. These devices can transduce chemical interaction phenomena into a signal that we can manage, compare and evaluate. Gas sensors are well-known chemical sensing devices, which may be integrated into personal healthcare (wound monitors, and breath analyzers) and security (toxic hazards and explosive detectors) systems. Moreover, they may also be used for environmental monitoring and for food-chain control. In particular, air pollution is recognized to be one of the most crucial issues for human health, and many efforts have been done by governments to reduce pollutant emissions. The main responsible of the degradation of air quality are identified as CO_2_, CO, NO_*x*_, Volatile Organic Compounds (VOCs), NH_3_ and small particulate matter (PM), i.e., PM_10_ and PM_2.5_ [9]. The detection of these toxic gases has become more and more essential for our own safety, thus it is necessary to have affordable and high performance gas detectors to measure these chemical compounds at sub-ppm level. Traditional gas sensing devices are based on semiconducting oxide materials, and can show excellent performances in terms of sensitivity and long-term stability. However, they are thermally activated and operate at very high temperature (200–600 °C), requiring a considerable amount of energy and making their use unsafe in explosive environment [6,10]. In many applications, there is therefore a huge need of sensitive gas sensors that can work at room temperature. 

Graphene and other carbon-based material are fundamentally and technologically appealing for many applications, including chemical sensing. They may operate at room temperature without requiring a dedicated heating element. However, they are chemically inert and they can only become active and interact with external atmosphere thanks to functionalization with some added molecules [11,12], which in turn results in losing some of the electronic and optical properties. In contrast, 2D TMDs exhibit a versatile chemistry while keeping some graphene features, potentially outperforming the latter in a real sensing environment. However, they are more resistant to chemical functionalization [13], and thus they may suffer the same selectivity issues of metal oxide materials. 

The goal of the present review is to summarize the advances and applications of molybdenum dichalcogenides as chemical sensors for air quality monitoring, evaluating the advantages and performances in the detection of typical air pollutant such as CO_2_, CO, NO_*x*_, VOCs and NH_3_.

## 2. Crystalline Structure and Synthesis Techniques

Molybdenum dichalcogenides, i.e., MoS_2_, MoSe_2_ and MoTe_2_, belong to the large family of layered transition metal dichalcogenides (TMDs) whose crystal structure results from the stacking of sheets of hexagonally packed atoms, with two chalcogen atom planes separated by a plane of metal atoms. Atoms forming this three-layer configuration are strongly packed together by covalent bonds, whereas each three-layer sheet is linked with the next one by Van der Waals bonds, much weaker than covalent bonds. These weak van der Waals forces between the sheets makes it easy to exfoliate thin layers from bulk material. Therefore, they share some properties with graphene, which, unlike TMDs, consists in only a single layer of *sp*^2^-bonded carbon atoms in hexagonal configuration [1]. 

The exfoliation of these materials into mono- or few-layers largely preserves their properties, making them ranging from insulators, semiconductors, true metals and even superconductors at low temperature, such as NbSe_2_ and TaS_2_ [7,14]. This peculiar structure leads to a high degree of anisotropy, with different (and usually significantly better) in-plane mechanical, thermal and electronic properties compared to out-of-plane ones [15], ranking for example MoS_2_ the most anisotropic 2D material after graphite [16]. 

Crystal phase is not unique for all TMDs: they exhibit a wide range of polymorphs depending on the phase of a single monolayer, which itself contains three layers of atoms (*X-M-X*), and on how monolayers stack together to form a bulk material. Therefore, a single TMD can be found in many different polymorphs, and its crystal structure is strongly related to its formation history. 

**Figure 3:**
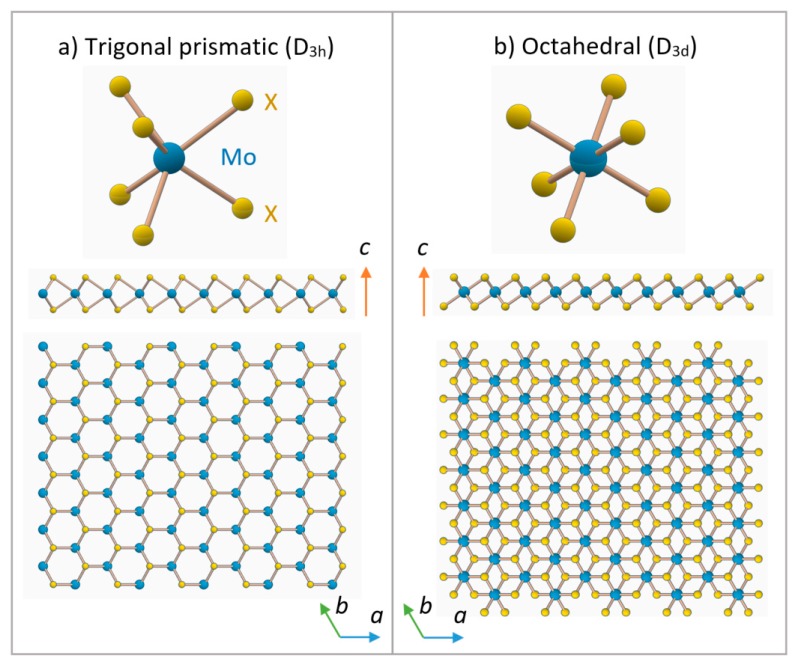
Trigonal prismatic (**a**); and octahedral (**b**) metal coordination, with respective c-axis and side sections, for Mo*X*_2_ materials. Mo atoms are in blue, chalcogenides (X) atoms in yellow. (**a**) Represents a monolayer with 1H crystal structure, while (**b**) a monolayer with 1T crystal structure. Atomic radii are not in scale.

Within a single monolayer, TMDs can exhibit only two polymorphs, directly related to metal coordination: trigonal prismatic (D_3h_ point group) or octahedral (D_3d_ point group), with a preferred structure depending on the specific combination of chalcogen and transition metal (Figure 3). Taken by themselves, these monolayers could be also named as 1H and 1T polymorphs, respectively, where “T” stands for trigonal and “H” stands for hexagonal. The digit refers to the number stacking layers (one in the case of a monolayer), which is also the number of *X-M-X* units forming the unit cell [7]. 

Bulk materials, instead, can be found in many different polymorphs. Most common ones are 1T, 2H and 3R (“R” stands for rhombohedral), which can easily be described as stacking sequence of monolayers. For example, 2H is characterized by |AbA BaB| stacking sequence, where capital and lower letters refer to chalcogen and metal atoms, respectively. The 3R crystal structure, instead, has a stacking sequence of |AbA CaC BcB|. 

Molybdenum dichalcogenides usually crystallize in 2H structure. Trigonal prismatic metal coordination is the most energetic favorable structure, and is the reason for the semiconducting behavior of these materials [17]. In some cases, synthetic MoS_2_ and MoSe_2_ could also crystallize as 3R triangular prismatic. For MoTe_2_, we have a phase transition at temperature higher than 815 °C from semiconducting 2H α-MoTe_2_ to metallic β-MoTe_2_, exhibiting a monoclinic structure with distorted octahedral coordination [18,19]. 

**Figure 4:**
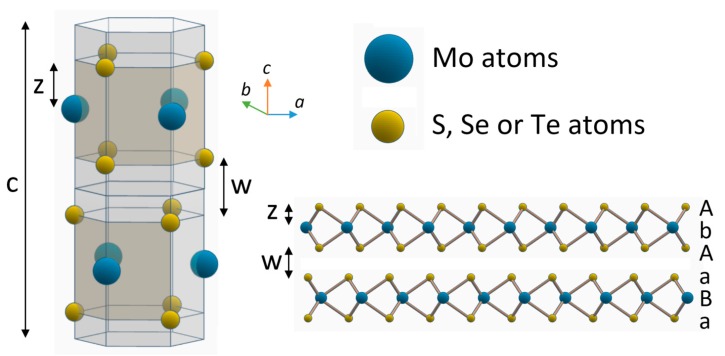
Crystal structure of 2H structured Mo dichalcogenides, with |AbA BaB| stacking sequence, where capital and lower letters refer to chalcogen and metal atoms, respectively.

A generic Mo*X*_2_ unit cell in 2H polymorph is displayed in Figure 4. A molybdenum atom plane is between two chalcogen planes, forming a monolayer. Two stacked layers are displaced respect to each other, having the metal atoms of the first layer directly above (along *c*-axis) the chalcogenides atoms of the second one, and vice versa. As previously described, layers are kept together by Van der Waals forces. The electronic structure of TMDs strongly depends on the coordination of the transition metal and the number of electrons in the *d*-orbital: for trigonal coordinated molybdenum, orbitals are fully occupied and Mo-dichalcogenides materials are thus semiconductors. Chalcogenides atoms, instead, have a minor effect on electronic properties. Lattice parameters increase with the increase of atomic number of the chalcogen, making the unit cell bigger, as reported in Table 1. At the same time, we can observe a gradual reduction of the indirect bandgap, for instance, due to the broadening of *d*-bands [20]. 

Electronic and optical properties not only depend on their chemical composition, but also on the thickness of these materials, and can be dramatically different. For example, bulk MoS_2_ shows an indirect bandgap of ≈1.3 eV, as reported in Table 1. However, an isolated MoS_2_ monolayer is a semiconductor exhibiting a direct bandgap of ≈1.8 eV due to quantum confinement effects, and thus enhancing significantly the photoluminescence compared to bulk material [21,22].

Quite interestingly, it is possible to intercalate alkali metals to induce a phase change in Mo-based TMDs. For example, we can turn semiconducting 2H-MoS_2_ into metallic 1T-MoS_2_ [23] by lithium or potassium intercalation [13,24,25,26], even if the 1T phase is not thermodynamically stable and switch back to the original 2H polymorph over time, even at room temperature [27]. Local phase transformations could potentially lead to hybrid metal-semiconductor 1T-2H, representing unique heterojunctions over a single homogeneous layer. 

**Figure 5:**
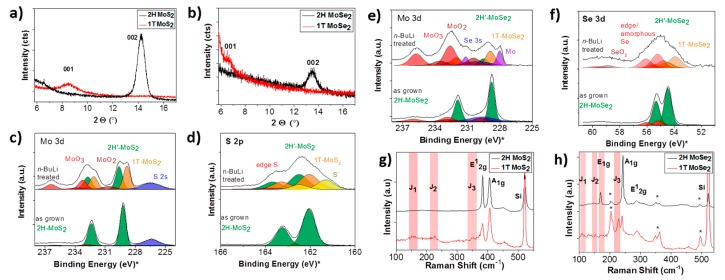
XRD pattern of: 1T and 2H MoS_2_ (**a**); and MoSe_2_ (**b**). XPS results for: Mo 3d (**c**); and S 2p (**d**) peaks of pure 2H and Li-intercalated MoS_2_. XPS results for: Mo 3d (**e**); and S 2p (**f**) peaks of pure 2H and Li-intercalated MoSe_2_. Raman spectra of: 1T and 2H MoS_2_ (**g**); and MoSe_2_ (**h**). Reprinted (adapted) with permission from [28]. Copyright (2016) American Chemical Society: Washington, DC, USA. .

The identification of crystallographic phases of Mo*X*_2_ compounds could be done by using standard spectroscopic techniques [28]. Figure 5 shows X-ray Spectroscopy (XRD) peaks of 2H and 1T MoS_2_ (Figure 5a) and MoSe_2_ (Figure 5b). The spectrum of 2H MoS_2_, for example, shows an intense peak at 14° related to (002) plane (ICSD code: 84183), indicating a *d*-spacing of ≈6.2 Å in line with cell parameters reported in Table 1. In Li-intercalated 1T structure, instead, (002) peaks is almost neglected, while we observe a new (001) reflection at ≈8.5°. 

X-ray Photoelectron Spectroscopy (XPS) is another technique for analyzing in detail the chemical state of 2H and 1T phases. In Figure 5 are reported fine XPS spectra of Mo 3d and S 2p for both 2H and 1T phases of MoS_2_ (Figure 5c,d) and MoSe_2_ (Figure 5e,f). By deconvolution of the peaks is possible to distinguish the contribution of both phases, estimating also relative concentrations [29,30]. Besides, Raman spectroscopy can easily identify the dichalcogenides polymorphs, but cannot provide accurate quantitative analysis. For example, 1T phase have symmetry differences which results in several additional vibration modes (*J*_1_, *J*_2_ and *J*_3_) not active in 2H (Figure 5g for MoS_2_ and Figure 5h for MoSe_2_) [26,27,30]. 

**Figure 6:**
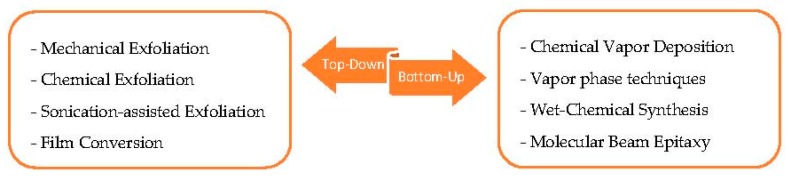
Bottom-Up and Top-Down approaches for the synthesis of thin 2D Mo*X*_2_ materials.

The chemical composition, phase structure, crystal quality, number of layers and edge morphologies have a strong effect on the performances of molybdenum dichalcogenides. However, the requirements depend on the proposed application: for high-end electronic devices it is necessary to fabricate high-purity and dopant-free materials, while for solar industry manufacturing cost is a key feature, and thus it is acceptable to have a certain amount of defects in the material [31]. Chemical sensing performances, in particular, are strongly affected by the synthesis technique used and the fabrication history of the samples. Across the years, many techniques have been developed for the fabrication of bulk and 2D thin film TMDs materials, which can be classified mainly as top-down and bottom-up approaches (Figure 6). 

Mechanical exfoliation was the earliest method introduced, back in the 1960s, to obtain 2D MoS_2_ [32]. It mainly consists in the removal of thin layers from the parent solid bulk thanks to subsequent Scotch adhesive tape transfers. Afterwards, the tape is placed on a target substrate and removed, leaving very thin layers (eventually monolayers) of materials. Although this technique is extremely cheap and could prepare high-quality monolayers, suitable to demonstrate high performances devices, it has some major drawbacks. Practical applications require fast production scale and bulk quantities of materials, which can hardly be obtained by Scotch tape technique. 

**Figure 7:**
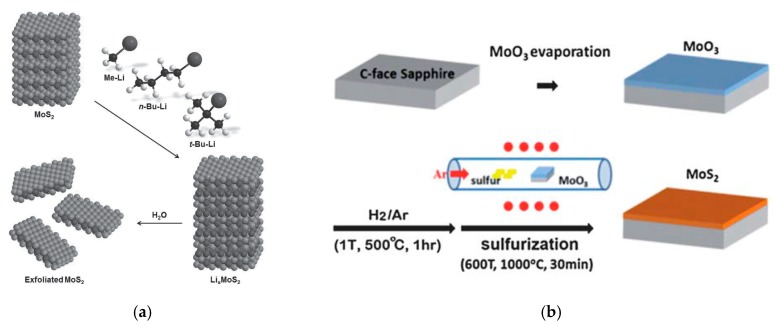
(**a**) Liquid exfoliation of MoS_2_ by using three different compounds: methyl lithium, *n*-butyl lithium and *tert*-butyl lithium. Reprinted with permission from [37]. Copyright (2015) Wiley-VCH Verlag GmbH: Hoboken, NJ, USA; (**b**) Schematic of conversion process from MoO_3_ layer to MoS_2_. Reprinted with permission from [41]. Copyright (2012) The Royal Society of Chemistry: London, UK.

TMDs, i.e., molybdenum dichalcogenides, are composed by uncharged layers kept together by Van der Waals forces. Contrary to charged-sheets solids such as perovskites, uncharged-layered solids cannot be chemically exfoliated easily [33]. Liquid alkali-atoms intercalation, such as lithium for example, enables the exfoliation of few-layers TMDs and eventually monolayers, and is more effective for devices mass production. By adding some lithium-based compounds, such as *n*-butyl lithium, in hexane solution it is possible to insert lithium atoms between every Mo*X*_2_ layer, as an intercalation agent. The reaction results in the formation of an intermediate Li_*x*_Mo*X*_2_ solid [34,35]. Layers are then separated by simple sonication in water, whereas lithium atoms are detached from the layers. Figure 7a reports an example of a typical liquid lithium exfoliation process for MoS_2_ [36,37]. This very effective technique can produce very high yield of monolayers, almost close to 100% rate [7]. However, it requires very long time (>3 days) and accurate control of the process to avoid the formation of undesired metal nanoparticles such as Li_2_S. Moreover, lithium exfoliation could lead to a phase changes from semiconducting 2H to metallic 1T, altering the electronic and optical properties of original molybdenum dichalcogenides materials [25,28,38], which can be restored afterwards by heat treatments [39]. An alkali-free alternative has been recently proposed by Coleman et al. [40], combining the advantages of liquid sonication-assisted exfoliation without causing distortions to the crystal structure. This latter technique was successfully employed to fabricate thin layers of molybdenum dichalcogenides; however, it has a much lower yield in the preparation of monolayers. 

Exfoliation methods produce quasi-2D materials, but require a certain amount of solid bulk crystals as source material, which have to be synthetized separately. Common techniques to prepare TMDs crystals are vapor-phase techniques such as chemical vapor transport (CVT) [42,43] and powder vaporization [44,45], mainly based on an evaporation-condensation process in a controlled environment. Other techniques used for the preparation of 2D thin films of molybdenum dichalcogenides include molecular beam epitaxy (Van der Waals Epitaxy (VDWE)) [46,47], metal organic chemical vapor deposition (MOCVD) [48,49] and direct metal or oxide conversion via exposure to a chalcogen vapor [41,50]. Figure 7b represents a schematic of the sulfurization of a thin MoO_3_ layer to obtain MoS_2_ on sapphire substrates [41]. A thin layer of desired thickness was deposited by thermal evaporation of molybdenum oxide powder on top of c-face sapphire, and then annealed in the furnace. Afterwards, samples were heated at high temperature together with a source of sulfur in inert atmosphere, resulting in oxide-dichalcogen conversion. This technique is very easy and leads to continuous TDMs films; however, it often results in undesired nanocrystalline structures. 

## 3. Molybdenum Disulfide (MoS_2_) Chemical Sensors

MoS_2_ is by far the most studied 2D material after graphene, and it could be considered as a prototypal TMD. MoS_2_ is an *n*-type semiconductor with highest direct and indirect bandgaps compared to other molybdenum dichalcogenides (Table 1). It has attracted huge attention in the last few years because of its excellent nanoelectronic, optoelectronic, and energy harvesting properties. Therefore, many research groups have started investigating the chemical sensing performances of devices based on this interesting material [51]. 

Especially suited for the fabrication of biosensors [52,53], MoS_2_ is attracting a strong interest in the field of chemical sensors for the detection on nitrogen dioxide (NO_2_), ammonia (NH_3_) and ethanol, among the most common pollutant gases. Nevertheless, Perkins et al. [54,55,56] investigated in detail the sensing properties of CVD MoS_2_ monolayers toward some laboratory chemicals and solvents, including triethylamine (TEA). Both simple conductometric [54] and FET [55] devices proved to be very sensitive to TEA and acetone, exhibiting almost no response to many other chemicals such as dimethylmethylphosphate (DMMP). In 2017, Li et al. [57] further improved TEA detection by fabricating a core–shell heterostructure, described as Au@SnO_2_/MoS_2_, by using a combination of different techniques. 

**Figure 8:**
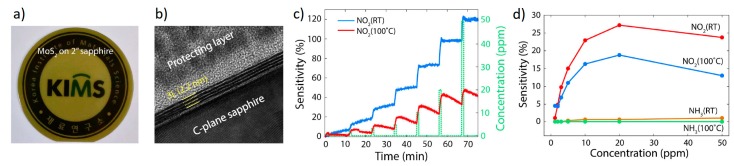
(**a**) Image of MoS_2_ semi-transparent film on the 2” sapphire substrate; (**b**) Cross-sectional Transmission Electron Microscopy (TEM) images, demonstrating that the synthesized MoS_2_ films consisted of three layers of MoS_2_; (**c**) Transient NO_2_ gas response at 1.5 to 50 ppm concentration, at operating temperatures of RT and 100 °C. The recovery rate was faster at 100 °C than at RT; (**d**) Comparison of responses to NO_2_ and NH_3_. Reprinted with permission from [58]. Copyright (2015) Nature Publishing Group: London, UK.

Cho et al. [58,59] were among the first proposing CVD grown MoS_2_ for gas sensing applications. In particular, systematic pressure control during the CVD process resulted in highly uniform three-layer MoS_2_ films on 2” wafer scale. Resistance responses (∆R/R) were investigated toward two common polluting gases: NO_2_ and NH_3_, at concentrations from 1.2 to 50 ppm (Figure 8). In particular, sensor resistance increased in presence of NO_2_ gas: NO_2_ acts as an electron acceptor, resulting in *p*-doping of the material. On the contrary, the resistance of the MoS_2_ sensing device decreased with the adsorption of NH_3_ gas molecules. In Fact, NH_3_ acts as an electron donor (i.e., *n*-doping) shifting the Fermi level of the MoS_2_ closer to the conduction-band edge. First-principles density functional theory calculations indicated that NO_2_ and NH_3_ molecules have negative adsorption energies (i.e., the adsorption processes are exothermic). Thus, NO_2_ and NH_3_ molecules are likely to adsorb onto the surface of the MoS_2_. Complete recovery of the baseline was hard to achieve: increasing the working temperature to 100 °C sped up the recovery substantially (Figure 8c). The charge transfer mechanism between the gas molecules and MoS_2_ was validated by theoretical calculations, indicating that the Fermi-level shift induced by the NH_3_ molecules is negligible. Interestingly, if SiO_2_ substrates were used instead of sapphire wafers, 2D MoS_2_ material switched its semiconducting behavior from *n*- to a *p*-type, due to doping caused by SiO_2_ dangling oxygen bonds [60]. Finally, same authors fabricated a bifunctional device able to work as gas sensor and as photodetector simultaneously [59]. Gas sensing measurements showed good response to low concentrations of NO_2_, although in nitrogen atmosphere. Moreover, the same measurements under 650 nm light illumination resulted in lower performances of the devices. 

**Figure 9:**
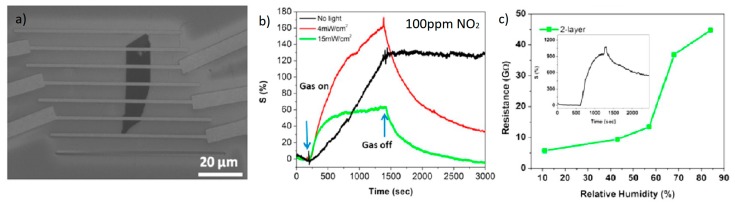
(**a**) SEM image of two-layer MoS_2_ transistor device; (**b**) Sensing behavior of five-layer MoS_2_ exposed to 100 ppm NO_2_ under green light illumination; (**c**) Resistance as a function of RH for two-layer MoS_2_ samples. Reprinted (adapted) with permission from [61]. Copyright (2013) American Chemical Society: Washington, DC, USA.

The possible reason of such a phenomenon was explained by Late et al. [61]. Authors prepared large-area MoS_2_ sheets ranging from single to five layers on 300 nm SiO_2_/Si substrates using the micromechanical exfoliation method, fabricating field effect transistor (FET) sensing devices that were assessed for gas-sensing performances to NO_2_, NH_3_ and humidity exposure, in different conditions of gate bias and light irradiation (Figure 9). Single layer devices had stability issues: they were not stable in air, and thus were not discussed. Interestingly, authors noticed that the five-layer MoS_2_ sample has better sensitivity (∆R/R) compared to that of the two-layer MoS_2_ sample. This phenomenon may be due to the different electronic structures caused by the different number of stacked layers. Electrical resistance in FET MoS_2_ can be tuned by gate biasing, which makes this material more competitive for gas sensing compared to, e.g., graphene. Thickest MoS_2_ device was more susceptible to the influence of gate bias. For all devices, however, recovery was not complete. To overcome this issue, researcher illuminated the samples with a green (532 nm) LED, instead of similar works UV light that could damage the structure due to the higher photon energy. Low power density irradiation slightly increased the response of devices compared to dark ones, but at higher power density the performances decreased significantly, as reported by [59] also. When there are too many photocarriers generated under strong illumination, not all the excited electrons/holes react with gas molecules. Moreover, it is possible that at high power density the desorption rate increases more than the adsorption rate because of the light-induced activation under irradiation. The humidity sensing performances of two-layer and five-layer MoS_2_ sensor devices were also investigated. Water vapor is considered an electron acceptor similar to NO_2_, so the resistance of MoS_2_ should increase with the relative humidity (RH). It resulted that below RH of 60% devices were almost insensitive to humidity. However, at higher humidity levels, the resistances change dramatically, especially for five-layer devices. Other research groups fabricating MoS_2_ devices by different techniques and with different morphologies also confirmed this important outcome [62,63,64]. 

A detailed analysis on a Schottky-contacted CVD monolayer MoS_2_ FET for the detection of NH_3_ and NO_2_ was reported by Liu et al. [65]. Authors believed that Schottky barrier modulation was the key factor for the significantly improved sensitivity, and that detection limit might be pushed to sub-ppb level by optimizing the features of the Schottky barrier.

Donarelli et al. [66] fabricated conductometric devices by dispersing liquid-exfoliated MoS_2_ and evaluated the performances toward NO_2_ in real air environment, investigating the effect of relative humidity on the response (R_air_/R_gas_) also. By controlling the annealing temperature, they were able to force a change in the semiconducting behavior of the material from *p*-type (150 °C) to *n*-type (250 °C). 

Although more difficult to achieve, chemical functionalization and the fabrication of heterostructures are effective techniques to tune the performances of functional devices. Lu et al. [67] proposed an interesting technique to include Au atoms in the MoS_2_ lattice. This approach firstly used a focused laser beam to locally unbound Sulfur atoms. Afterwards, substrates were immersed in AuCl_3_ solution, forcing the anchoring of Au nanoparticles to these active sites. Samples were then characterized as starting brick for surface enhanced Raman scattering (SERS) devices.

A more conservative approach was illustrated by Baek et al. [68]. Authors put Pd nanoparticles by simple thermal evaporation on top of commercially available MoS_2_ sheets deposited by drop casting, to fabricate a resistive hydrogen sensors. The thickness of the Pd nanoparticle layer was controlled from 1 nm to 7 nm. At low Pd thickness functionalization resulted in an increase of the response to 1% H_2_ keeping the same baseline resistance of devices. On the contrary, at highest Pd concentration the sensing layer suddenly became metallic. The reason is that Pd nanoparticles formed a continuous film, completely changing the electronic properties of devices. 

Apart from 2D crystalline mono and few-layers devices, some research groups have studied different morphologies of MoS_2,_ which may exhibit different sensing properties compared to both its bulk and 2D counterparts. Liu et al. [69] proposed a MoS_2_/Si *pn* junction devices for ammonia sensing, fabricated by magnetron sputtering from a MoS_2_ target, with a peculiar vertical structure instead of the more common planar one. This device was able to sense high concentration of ammonia, although with a low response (∆G/G ≈19.1%@200ppm NH_3_). At the same time, authors investigated the hydrogen sensing performances of this MoS_2_/Si device [70]. In presence of 30% relative humidity in air, proposed device was able to detect 5000 ppm of H_2_ with a response (∆I/I) of 15.4%. Moreover, controlling the relative humidity during measurements, authors asserted that water molecules have no effect on electrical properties of the material, but they compete with hydrogen molecules occupying the same surface sites. 

Yan et al. [71] mixed ZnO nanoparticles with MoS_2_ nanosheets grown by hydrothermal methods, and evaluated the gas sensing performances of conductometric devices toward some VOCs including ethanol. Optimal working temperature of pure and ZnO-coated devices were detected at 240 °C and 260 °C, respectively, resulting in a response (R_air_/R_gas_) of the latter equal to 42.8@50ppm of ethanol. Response to other VOCs such as methanol was significantly lower, making the devices partially selective to ethanol. 

Sponge-like structures of MoS_2_ were prepared by Yu et al. [72] by hydrothermal technique and integrated into a conductometric device. They identified 150 °C as optimal sensing temperature for NO_2_ detection, and measured a maximum response (R_gas_/R_air_) of 78% to 50 ppm of NO_2_, diluted in air. The material behaves like a *p*-type semiconductor, and, even though the recovery of the baseline was thermally assisted in vacuum (650 °C for 1 h), reported response was extremely stable showing a maximum difference of ≈1% during one week.

Another porous structure was proposed by Dwivedi et al. [73]. *p*-type silicon wafer was etched by electrochemical anodization to obtain a porous silicon substrate, on which metallic molybdenum was deposited on top by magnetron sputtering. The film was then oxidized to obtain MoO_3_, which was converted to *n*-type MoS_2_ by sulfurous film conversion as described in [41], forming a *p*-*n* junction. Porous MoS_2_ samples were characterized for the detection of methanol, ethanol, acetone and other VOCs, using nitrogen as carrier gas. Sensor response ∆R/R was quite low at 1 ppm, but porous samples performed over five times better than flat MoS_2_. The enhancement may be attributed to increased surface area, and to the barrier effect of *p*-*p* junction between porous and flat silicon. Moreover, the response was stable over more than two months. 

Quantum dots (QDs) of MoS_2_ and graphene oxide (GO) were mixed together to create a hybrid sensing material by Yue et al. [74]. GO and MoS_2_ powders were processed to obtain QDs liquid solution with G/M mass ratios of 1:1, 3:1 and 5:1, and then characterized to confirm the morphological, optical and gas sensing properties. Hybrid G/M QDs performed better than their counterparts alone did in detecting both NO_2_ and NH_3_. In particular, 3:1 device showed the highest response (∆R/R) thanks to charge transfer mechanism from adsorbed molecules and *p*-type QDs. All samples, however, exhibited a drift during recovery in N_2_. Interestingly, illumination of samples with 532 nm light source did not influenced significantly the performances of devices, and was due to local heating. For monolayer MoS_2_, the photo-thermoelectric effect is more dominant to the photocurrent than photoexcited electron–hole pairs across the Schottky barriers [75]. 

Electrohydrodynamic (EHD) printing process was realized by Lim et al. [76] to deposit a uniform distribution of exfoliated MoS_2_ flakes on desired substrates, to prepare conductometric chemical sensors. 

Yan et al. [77] demonstrated the ability of MoS_2_ nanosheets in preventing the aggregation of dispersed SnO_2_ nanoparticles. The presence of MoS_2_ allowed decreasing the optimal working temperature from 340 °C to 280 °C, exhibiting a response R_gas_/R_air_ of ≈50 to 50 ppm of ethanol. However, Cui et al. [78] showed that decorating SnO_2_ nanocrystals could stabilize MoS_2_ nanosheets in air. Quite surprisingly, the combination of *n*-MoS_2_ and *n*-SnO_2_ fabricated by wet chemistry resulted in a *p*-type behavior of the heterostructure, and was able to dramatically enhance the stability, reproducibility and sensibility of the response and devices, in particular for NO_2_ detection. A similar switch in the semiconducting behavior of MoS_2_-based heterojunctions was detected by Zhao et al. [79] in well-aligned MoS_2_-decorated TiO_2_ nanotubes. Finally, Zhou et al. [80] reported in 2017 the synthesis of hybrid rGO/MoS_2_ composites via wet chemistry, and a comparison of NO_2_ sensing performances of pure rGO and rGO/MoS_2_. Baseline resistance of the latter was one order of magnitude higher than rGO device, and the response of the sensor was doubled. The effect of temperature, relative humidity and stoichiometry of the material on the sensing properties, including stability of the devices, was also discussed. 

Table 2 reports a summary of chemical sensing MoS_2_ devices found in literature.

## 4. Molybdenum Diselenide (MoSe_2_) Chemical Sensors

**Figure 10:**
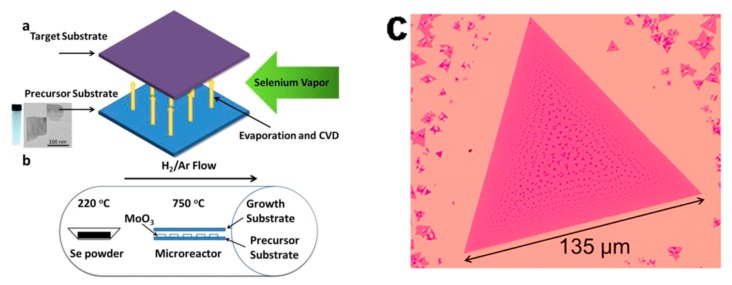
(**a**) Schematic of CVD process between the seed and target substrates, where selenium reacts with MoO_3_ nanosheets to form MoSe_2_ layers on the top substrate; (**b**) schematic of furnace setup; and (**c**) typical optical images of monolayer triangles, with some small bi-layered domains in darker color. (**a**,**b**) Reprinted with permission from [85]. Copyright (2015) Wiley-VCH Verlag GmbH. (**c**) Reprinted with permission from [86]. Copyright (2014) American Chemical Society: Washington, DC, USA.

Atomically thin MoSe_2_ is another good candidate for the fabrication of nanosized electronic devices [82]. It has a low bandgap (1.1–1.5 eV) and an appreciable mobility (≈50 cm^2^ V^−1^s^−1^), which allow the fabrication of switchable transistors and sensitive photodetectors devices. Thanks to its optical properties, it also proved to be a good candidate for the fabrication of fiber-optic humidity detectors [83]. Chemical vapor deposition (CVD), briefly illustrated in Figure 10, is the most common technique to prepare very thin flakes directly on target substrates [84,85,86], for the fabrication of functional devices. Molecular bean epitaxy (MBE) was also explored [87]. While MoSe_2_ electronic and optical performances have been largely investigated, few reports exist on its chemical sensing behavior.

**Figure 11:**
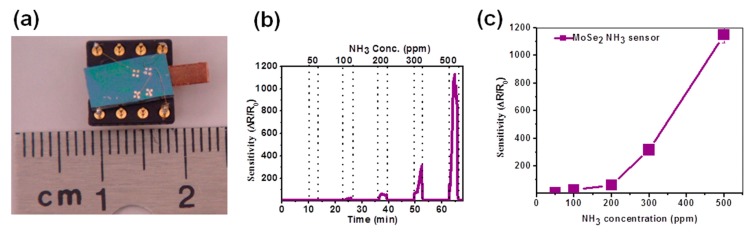
(**a**) Optical image gas of MoSe_2_ sensor device; (**b**) NH_3_ sensing response as function of gas concentration; and (**c**) linear plot of sensitivity of MoSe2 gas sensor device as a function of NH3 gas concentration (ppm). Reprinted with permission from [88]. Copyright (2014) AIP Publishing LCC: Melville, NY, USA.

The first reported MoSe_2_ gas sensing device was presented by Late et al. in 2014 [88]. They mechanically exfoliated a flake of MoSe_2_ from a bulk crystal, which was deposited on SiO_2_/Si substrate. Electrodes were deposited by electron beam lithography (EBL). Although authors fabricated a field effect transistor, it was used as a simple conductometric device, in which gate bias was put to zero. Sample was exposed to different concentration of ammonia (50–500 ppm) at room temperature, followed by a recovery in Ar gas to avoid oxygen doping of the sensing material. Response, defined as ∆R/R, was extremely high, exceeding 1000 in presence of 500 ppm of NH_3_ (Figure 11c). However, it reduced quickly as the gas concentration decreased, and it showed an irregular dynamic behavior at low concentrations (Figure 11b). Authors detected fast response and slightly longer recovery time, due to the strong adsorption of ammonia molecules that are difficult to remove from the surface of the sensing material. 

**Figure 12:**
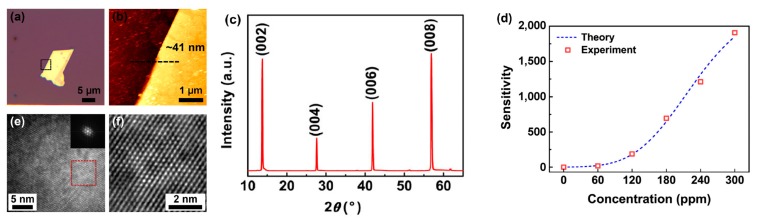
(**a**) Optical image; (**b**) Atomic Force Microscopy (AFM) image; (**c**) XRD pattern; (**e**) Fast Fourier Transform (FFT); and (**f**) inverse FFT image of CVD-grown multilayer MoSe_2_ flake. The thickness of the MoSe_2_ flake is about 41 nm. (**d**) Sensitivity vs. NO_2_ concentration, where the dashed blue line is based on a model, and the red square symbols represent the data from the experiments. Reprinted with permission from [89]. Copyright 2017 Springer-Verlag: Berlin/Heidelberg, Germany.

Baek et al. characterized CVD grown MoSe_2_ flakes towards NO_2_, in both diode and FET configuration [89], transferred on SiO_2_/Si substrate by mechanical exfoliation (Figure 12). In FET configuration, this sensor exhibited high response (∆I/I) resulting in more than 1900 at very high NO_2_ concentration of 300 ppm, using N_2_ as carrier and recovery gas. This amount of NO_2_ by far exceeded any regulated threshold (EU regulation: <0.1 ppm for 1-h exposure [90]): it is higher than the IDLH value (20 ppm), potentially being lethal to living beings [91]. High sensitivity of this gas sensor is attributed to changes in the gap states near the valence band induced by the NO_2_ gas absorbed on the MoSe_2_. Moreover, proposed device exhibited fast response times and rapid on-off switching. Authors also performed quantum transport simulations to verify the I-V characteristics, which resulted in a very good agreement with experimental data. 

**Figure 13:**
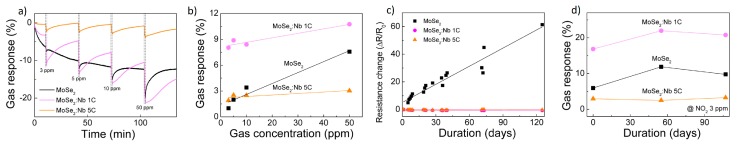
(**a**) Dynamic response of the Nb-doped MoSe_2_ devices at NO_2_ concentrations ranging from 3 to 50 ppm; and (**b**) responses of the three devices as a function of the NO_2_ gas concentration. (**c**) Resistance change data over 120 days: Nb-doped MoSe_2_ devices showed negligible resistance change, differently from MoSe_2_ devices; (**d**) Comparison of the long-term stability of the three devices with respect to the gas sensing response. All gas-sensing tests were performed at an operating temperature of 150 °C. Reprinted with permission from [92]. Copyright (2017) American Chemical Society: Washington, DC, USA.

Although functionalization of TMDs materials is quite challenging due to their intrinsic nature, Choi et al. were successful in doping MoSe_2_ with Nb atoms [92]. They fabricated Nb-doped MoSe_2_ conductometric devices on sapphire substrates by a combination of different techniques for NO_2_ gas sensing. Firstly, MoO_3_ thin film (3 nm) was deposited by thermal evaporation, followed by one or five plasma enhanced atomic layer deposition cycles of Nb_2_O_5_ (PEALD) on top of MoO_3_ layer, with a thickness of 0.8 nm and 1.6 nm, respectively. Thermal selenization was carried out by film conversion technique similar to the one described in [41]. Niobium doping resulted in a significant decrease of electrical resistance, up to four order of magnitude, due to the metallic characteristics of NbSe_2_ [93]: five-cycle Nb samples experienced a doping-induced semiconductor-metal transition. Different from previous works, measurements were carried out at 150 °C in both N_2_ and air, confirming the *p*-type behavior of the material. Slightly doped MoSe_2_ was able to achieve the highest response (∆R/R), however all devices suffered a significant drift of the baselines (Figure 13a,b). Interestingly, the authors took in consideration the stability of the device over time. After a period of 4 months, Nb-doped devices exhibited small electrical resistance changes. On the contrary, pure MoSe_2_ devices suffered from a strong oxidation that resulted in a 60% change of resistance (Figure 13c). The response to NO_2_ was affected similarly (Figure 13d), confirming the enhancement of Nb-doping not only on the sensitivity but also on the stability of the devices.

Table 3 reports a summary of chemical sensing MoSe_2_ devices found in literature.

## 5. Molybdenum Ditellurite (MoTe_2_) Chemical Sensors

Molybdenum ditellurite (MoTe_2_) has a very small bandgap of only 0.8–1.1 eV depending on the morphology, lower than many TMDs including other molybdenum dichalcogenides [94]. It has a strong absorption throughout the entire solar spectrum, and thus it has been adopted as electrode material in photovoltaic cells [95]. Thanks to its good room temperature carrier mobility (up to 200 cm^2^ V^−1^ s^−1^) [96], and to the possibility to tune the growth to obtain *n*- and *p*-type semiconductors, it is well suited for the fabrication of transistors and LED [97,98,99]. Moreover, it is possible to force a phase transition from semiconducting α-MoTe_2_ to metallic β-MoTe_2_, leading to the formation of interesting nanosized Schottky junctions [19,100] and showing a unique potential in phase-transition devices [101]. Finally, it has a low thermal conductivity [102] and may show a superconductive behavior [103,104]. However, as reported in the Introduction, ditellurite materials are the least common class of TMDCs, and very few reports exist on the application of MoTe_2_ as chemical sensors. 

This material is usually synthetized by chemical vapor transport [99], exfoliation [17], CVD [105,106] or MBE [107], and then transferred on destination substrates used for the preparation of functional devices. The most common configuration is the field effect transistor, in which a MoTe_2_ flake is deposited on source and drain electrodes on a passivated silicon substrate. Unfortunately, very thin layers of MoTe_2_ are not stable in air, and readily oxidize to MoO_3_ and TeO_2_ keeping the two dimensional morphology [107,108]. However, oxidation process is not only due to the presence of oxygen itself. The introduction of pure oxygen in a low vacuum environment, even at high temperature, is not enough to oxidize the material: possibly water or other atmospheric oxidant are involved too. This from one side confirms the sensibility of the material toward the surrounding atmosphere, but from the other affects the stability of MoTe_2_ chemical sensor devices, which potentially have a limited lifetime and may exhibit a gradual loss of performance. 

Lin et al. [109] were the first in 2015 investigating the possible application of MoTe_2_ in field effect transistors for environmental sensing. They synthetized semiconducting α-MoTe_2_ by mechanical exfoliation of a bulk crystal grown by chemical vapor transport, which was deposited on SiO_2_/Si [99]. Bulk silicon was connected as gate contact, while two thin Ti/Au contacts acted as source and drain electrodes. Exposition of the devices to ambient air resulted in a reduction of carrier mobility by half compared to the original values in vacuum, and in an increased hysteresis of the transfer characteristics due to the adsorption of oxygen molecules. Quite interestingly, authors demonstrate that low frequency electronic noise of these devices depends on MoTe_2_ conductive channel itself rather than on contact barriers, and that this noise is affected strongly by the interaction with surrounding atmosphere. However, no performance evaluations are reported. 

A functional FET device based on MoTe_2_ was presented, instead, by Feng et al. in 2016 [110], starting from an exfoliated flake deposited on passivated silicon substrate, similarly to [107]. Authors could tune the semiconducting behavior of the material from *p*- to *n*-type by thermal annealing, and evaluated the performances of these *p*- and *n*-type devices toward NO_2_ and NH_3_, respectively, some typical air pollutant. Both devices were highly dependent on gate bias voltage, with zero bias as optimal condition for sensing measurement (sub-threshold region) [111]. In these conditions, *p*-MoTe_2_ exhibited a response (defined as ∆G/G%) of 140% toward 100 ppb of NO_2_, exceeding 1000% toward 1 ppm, at room temperature. In the same experimental condition *n*-MoTe_2_, instead, resulted in a response of ≈30% toward 2 ppm of NH_3_. Response and recovery time at zero gate bias were extremely fast, recovering the baseline in less than 10 min for both devices and gases. On the contrary, the application of a positive or negative bias always resulted in lower performances. 

**Figure 14:**
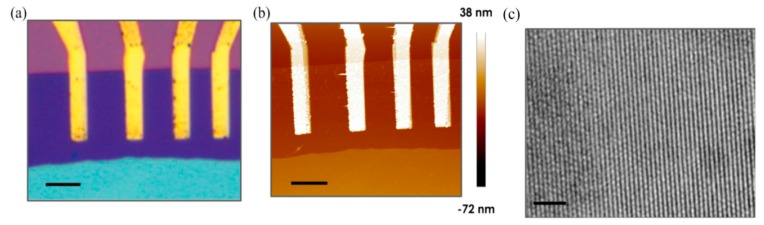
(**a**) Optical microscope image of the MoTe_2_ field-effect transistor (FET). Scale bar is 5 μm; (**b**) Atomic force microscopy (AFM) topography image of the MoTe_2_ FET. Thickness of the MoTe_2_ is 3.4 nm. Scale bar is 5 μm; (**c**) High-resolution transmission electron microscopy (TEM) image of a typical exfoliated MoTe_2_ film. Scale bar is 2 nm. Reprinted from [112], MDPI, Basel, Switzerland.

The same authors achieved a further improvement to NH_3_ detection by illuminating *n*-type MoTe_2_ FET devices with a continuous light source [112]. Optical, atomic force microscopy (AFM) and transmission electron microscopy (TEM) pictures are reported in Figure 14a–c, respectively. The effect of both photon energy (wavelength) and intensity was investigated, always resulting in higher performances of illuminated devices compared to the one in dark. As demonstrated by previous investigations, zero gate bias was applied during electrical measurements. 

**Figure 15:**
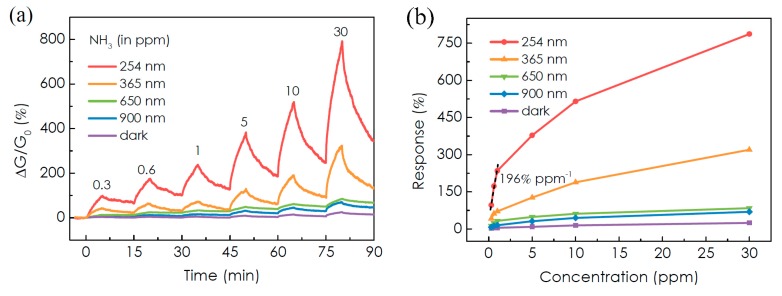

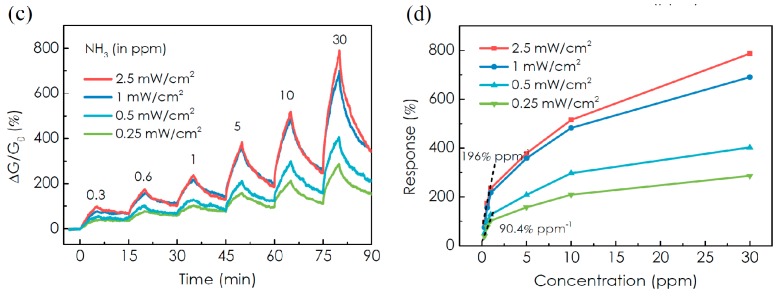
(**a**) Real-time conductance change of the MoTe_2_ sensor upon exposure to different concentrations of NH_3_ in the dark and under light illumination; (**b**) calculated response and calibration curves of the MoTe_2_ sensor as a function of wavelength (photon energy); (**c**) real-time conductance change of the MoTe_2_ sensor upon exposure under 254 nm UV light illumination; and (**d**) calculated response and calibration curves of the MoTe_2_ sensor as a function of UV light intensity. Reprinted from [112], MDPI, Basel, Switzerland.

The response of the MoTe_2_ sensor toward NH_3_ is enhanced remarkably under light illumination from near-infrared (900 nm) to UV region (254 nm), keeping the same light intensity (2.5 mW/cm^2^), as reported in Figure 15a,b. Even though MoTe_2_ has a small bandgap compatible with near-infrared energy, 254 nm UV light is the most effective in enhancing the response of the FET device. In particular, it ranges from 4% (300 ppb) to 25% (30 ppm) under dark condition, whereas it range from 100% to 790% under UV light, 50 times higher than dark response. UV illumination has the side effect of forcing a behavior change from *p*- to *n*-type, due to the removal of residual O_2_ molecules from MoTe_2_ flake. Oxygen removal is deemed the reason of enhanced response of the devices, releasing more surface active sites. UV light intensity has a similar effect: applying an intensity from 0.25 mW/cm^2^ to 2.5 mW/cm^2^ the response to 30 ppm of NH_3_ increases from 290% to 790% (Figure 15c,d). 

Table 4 reports a summary of chemical sensing MoTe_2_ devices found in literature.

## 6. Conclusions

The properties of bulk TMDs, especially molybdenum dichalcogenides, are diverse, and they get even more interesting at nanoscale level in form of 2D thin layers. They share the same facile synthesis techniques, mainly based on exfoliation of bulk material, which are reminiscent of graphene and other 2D materials. However, the chemistry of Mo*X*_2_ compounds offers opportunities for going beyond graphene and opening up new fundamental and technological pathways for inorganic 2D materials. 

Metal oxide materials are still the state-of-the-art for the fabrication of environmental chemical sensors, especially in form of nanostructures such as nanowires, nanoparticles and nanotubes, thanks to their superior performances that are the results of years of research activities. However, they have some limitations which are pushing the research to look for alternative sensing materials. Among these, emerging TMDs play an important role, but are not the only protagonists [113]. Black Phosphorous (BP), for instance, has proven to be quite sensitive and selective to NO_2_ gas [114]. MoS_2_, instead, is sensitive to many other different chemical compounds also [115], and thus is not favorable for the fabrication of highly selective devices, which is also one of the strongest limits of metal oxides. Graphene has much lower performances for NO_2_ and in general for chemical sensing, therefore cannot be used if sensitivity is a requirement. 

All these extremely innovative materials, however, pose challenges when their integration into sensing devices is performed at industrial scale. Mechanical exfoliation is not feasible to produce large amount of devices in a reproducible way, and liquid exfoliation has some limitations too. Chemical vapor deposition or thin film conversion are better suited, but the high temperatures required could be incompatible with traditional silicon processing. 

According to the very first reports, among molybdenum dichalcogenides, MoTe_2_ appears as the most exciting material for gas sensing applications, due to its extreme sensitivity to the surrounding atmosphere. Despite the very interesting results reported in literatures, in real field applications things could be different from expected. In many works, for example, performances were evaluated using nitrogen or argon as carrier gas instead of synthetic air: due to the lack of oxygen, these sensing measurements are not truly representative of the performances of the devices, avoiding the degradation of the material due to progressive oxidative processes. Therefore, it is very difficult to have the complete picture of the performances of these materials in terms of sensibility and stability over time, in particular for MoSe_2_ and MoTe_2_ which are not investigated properly yet. Their spontaneous degradation in atmosphere is one of the biggest limiting factor for the use of these materials in commercial devices.

Research groups who have dealt with stability issues stress that, in many cases, recovery of devices was not complete, even for MoS_2_ that is the most studied TMD material. In particular, in the case of environmental sensors, it is difficult to release NO_2_ and NH_3_ molecules from material. Thermally assisted recovery could be an option, but we lose one of the biggest advantage of these materials: the possibility to operate at room temperature. The combined use of light illumination, with tuned wavelength and intensity, proved to be a valid alternative to enhance the performances of the devices, but could induce modification in the structure of the materials or make them degrade faster. Another option is the chemical functionalization of the surface or even integration of other 2D materials, such as BP or graphene. This may also result in the stabilization of these Mo*X*_2_ in presence of oxidizing atmosphere, and further enhance the selectivity among target chemical species. However, research activities on TMDs material are growing exponentially; therefore, it is reasonable to expect many scientific breakthrough in the next few years, which will be potentially able to revolutionize gas sensor market. 

## Figures and Tables

**Table 1:** Cell and structural parameters and measured bandgaps of 2H polytype Mo dichalcogenides [18,19,20].

	MoS_2_	MoSe_2_	MoTe_2_
a [Å]	3.160	3.299	3.522
c [Å]	12.294	12.938	13.968
2z [Å]	3.172	3.338	3.604
w [Å]	2.975	3.131	3.380
c/a [Å]	3.891	3.922	3.966
Indirect Bandgap [eV]	1.29	1.10	1.00
Direct Bandgap [eV]	1.78	1.42	1.00

**Table 2:** Summary of MoS_2_ gas sensing devices, target chemical compounds and performances. RT stands for room temperature (^1^ in N_2_ atmosphere; ^2^ in Ar atmosphere; ^3^ in presence of a relative humidity (RH) of 30%; ^4^ in presence of a relative humidity (RH) of 45%).

Ref.	Material	Growth Technique	Device Type	Gas and Temperature	Performances
[58]	MoS_2_	CVD	Resistive	NH_3_-RT NO_2_-RT	<2%@20ppm ≈27%@20ppm
[59]	MoS_2_	CVD	Resistive	NO_2_-RT	≈120%@1ppm ^1^
[61]	MoS_2_	Mechanical Exfoliation	FET	NH_3_-RT NO_2_-RT	≈50%@200ppm ^1^ ≈400%@20ppm ^1^
[65]	MoS_2_	CVD	FET	NH_3_-RT NO_2_-RT	≈50%@200ppm ^2^ ≈50%@100ppb ^2^
[66]	MoS_2_	Liquid Exfoliation	Resistive	NO_2_-200 °C	5.8@1ppm
[68]	MoS_2_ + Pd NPs	Drop Casting + Evaporation	Resistive	H_2_-RT	≈35%@1%
[69,70]	MoS_2_/Si	Magnetron Sputtering	Resistive *pn* junction	NH_3_-RT H_2_-RT	≈300%@50ppm 15.4%@5000ppm ^3^
[71]	MoS_2_ + ZnO	Hydrothermal	Resistive	Ethanol-260 °C	42.8@50ppm
[72]	MoS_2_	Hydrothermal	Resistive	NO_2_-150 °C	78%@50ppm
[73]	MoS_2_ Porous	Sputtering + Film Conversion	Resistive	Ethanol-RT	≈2%@1ppm ^1^
[74]	MoS_2_/GO QDs	Exfoliation + Sonication	Resistive	NO_2_-RT NH_3_-RT	≈35%@10ppm ^1^ ≈20%@10ppm ^1^
[76]	MoS_2_ Flakes	Sonication	Resistive	NO_2_-100 °C CNH_3_-100 °C	≈10%@10ppm ^1^ ≈35%@10ppm ^1^
[77]	SnO_2_@MoS_2_	Hydrothermal	Resistive	Ethanol-280 °C	≈50@50ppm
[78]	SnO_2_@MoS_2_	Hydrothermal	FET	NO_2_-RT	≈28%@10ppm
[79]	MoS_2_@TiO_2_	Hydrothermal	Resistive	Ethanol-150 °C	14.2@100ppm ^4^
[80]	MoS_2_/rGO	Hydrothermal	Resistive	NO_2_-RT	≈60%@2ppm
[81]	MoS_2_	Liquid Exfoliation	FET	NO_2_-RT	≈11%@1ppm ^1^

**Table 3:** Summary of MoSe_2_ gas sensing devices, target chemical compounds and performances. RT stands for room temperature (^1^ in Ar atmosphere; ^2^ in N_2_ atmosphere).

Ref.	Material	Growth Technique	Device Type	Gas and Temperature	Performances
[88]	MoSe_2_	Mechanical Exfoliation	Resistive/FET	NH_3_-RT	≈200@200ppm ^1^
[89]	MoSe_2_	CVD + Mechanical Exfoliation	Resistive/FET	NO_2_-RT	1907@300ppm ^2^
[92]	Nb-doped MoSe_2_	ALD + Film Conversion	Resistive	NO_2_-150 °C	8%@3ppm ^2^

**Table 4:** Summary of MoTe_2_ gas sensing devices, target chemical compounds and performances. RT stands for room temperature. * in N_2_ atmosphere.

Ref.	Material	Growth Technique	Device Type	Gas and Temperature	Performances
[109]	MoTe_2_	Mechanical Exfoliation	FET	Air	-
[110]	MoTe_2_	Exfoliation	FET	NO_2_-RT NH_3_-RT	140%@100ppb * ≈30%@2ppm *
[112]	MoTe_2_	Mechanical Exfoliation	FET + Light Illumination	NH_3_-RT	100%@300ppb *
